# Willing to Do the Math: An Interview with David Botstein

**DOI:** 10.1371/journal.pgen.0020079

**Published:** 2006-05-26

**Authors:** Jane Gitschier

David Botstein's name came up twice when the *PLoS Genetics* editors tossed around ideas for potential interviewees, and with good reason. First, he is now director of an exciting development on the Princeton University campus, the Lewis-Sigler Institute for Integrative Genomics, which weaves the physical, computational, and biological sciences into a cohesive endeavor. Second, his scientific discoveries have run the gamut of organisms, from phage P22 to yeast to humans. And third, David is no shrinking violet; he has a deeply held opinion about everything, and he isn't afraid to voice it.

For those of you who are familiar with David, these words will likely fly off the page. There is little need to ply him with pre-formed questions; once you get him going, he is a verbal tsunami. His zeal is legendary, and his delight in people and ideas is palpable.

I met David at his office in the Carl Icahn Laboratory on a chilly February morning after the snow had melted. The building is the elegant design of Rafael Vinoly. Its airy entranceway, which spans the length of the curved glass façade, embraces a small café, a cylindrical seminar hall, and, jarringly, the 30-year-old lead-clad prototype of a Frank Gehry house, which David aptly refers to as the “armadillo.” Double-helical shadows are cast by latticed aluminum pillars that hug the portico's arc, and altogether too stylish, white, Tom Vac and Pierre Paulin “Orange Slice” chairs populate the space. Yet tucked into a corner were six dowdy Revcos and an ice-o-matic, signaling that this building is not just a pretty face.

I powered up my fancy new digital Marantz recorder, stoked with a high-capacity flash card, and pushed the record button. Here follow excerpts from what proved to be the first in a double-header interview.

**Jane Gitschier:** Let's start with the Lewis-Sigler Institute. What are you trying to do here?

**David Botstein:** Actually, I came here to do something about science education, an experiment, if you like. As you know, I was at MIT [Massachusetts Institute of Technology] for many years and taught undergraduate and graduate courses, and was director of the graduate program. Beth Jones and I invented the whole project lab system. We were instructors at that time [1969], a non-tenure track appointment. You worked in somebody's lab, but you did teaching.

**JG:** Whose lab were you in?

**DB:** I was with Maury Fox. Beth brought yeast to Boris's [Magasanik] lab. I brought P22 to Fox's lab. We weren't randomly chosen to do this. I think the idea was that they brought people who were particularly good experimentalists to teach these lab courses.

We were given this really awful course to teach, and we made a proposal to teach it in a better way, which involved major changes in the curriculum, and they [the older faculty] went along with it. And in fact, I got a job [as Assistant Professor] out of it.

There is something about teaching that makes you a better researcher. I know this is very countercultural wisdom, but I believed it all along. Luria, Magasanik, and Levinthal all believed it. Levinthal and Luria both had a very strong influence on me in this regard.

That's one part of the story!

The other part of the story is that through the Academy [National Academy of Sciences], I became very interested in the question of why it is that there are fewer and fewer kids in America who are interested in biology. And through the Academy studies, it became clear from the statistics that the total number of kids interested in any kind of science had been falling for the last 30 years. And the conventional wisdom is that it is all about K–12 education.

I don't believe that for a second.

**JG:** And why is that?

**DB:** I'll tell you the argument. There are three arguments why this is not right.

One is that it is a mistake to think that the high schools were ever so good. There is zero argument to say that they have gotten any worse. If you ask about the absolute number of kids who get calculus in high school, it has risen 10- or 20-fold in the last 30 years. When I went to college in the late 50s, I actually did have advanced placement, but it was rare!

Second argument is that if you look at the outcomes of kids who get degrees in science and then go to graduate school—this is actually a Tom Cech study published in the *Daedalus* just before he became head of the Hughes—and you ask where the graduate students come from, it turns out that small colleges, like Swarthmore, who draw from exactly the same pool as the big universities like Princeton, Harvard, and Stanford, are many-fold better at motivating students to become professional scientists. It's something like 5-fold.

Third argument is that who was teaching whom in the 50s and 60s was very different [than now]. I had biochemistry from Konrad Bloch. This was Konrad Bloch before he got the Nobel Prize. He had nothing better to do than teach 120 kids biochemistry. He did it very well. He took it very seriously. He did not go to the dean and say, “Oh, I have this grant and I'm about to win a Nobel Prize and therefore I should be free to do research.”

He understood, as I think we all understood, that there is an organic connection between teaching and research. So I came with all this baggage. And Ira Herskowitz, and basically the MIT school, had that baggage. You remember what it was like! Luria taught 7.01 [introductory biology]; Boris taught microbiology. Gene Brown had 7.05 [biochemistry]. The leaders were leading from the front!

For various reasons, there were issues at Stanford, and I was motivated at least to look at my alternatives. I had been talking on and off with Shirley [Tilghman], because this institute was started and Shirley was going to be the director, and then they made her president [of Princeton]. And she had incorporated the idea, which I think was really very smart, that there was already at Princeton an unusually close relationship between the molecular biologists and the physicists.

There came a moment when I called Shirley and said I had ideas about what could been done with such a place. It was a substantial departure from what she had intended. I submitted myself to the formal process and tried to convince everybody to do what we're doing—which was to say, “Let's see if we can do something for a subset of students who, I believe, are underserved in the university.” Students who are not going to medical school, necessarily, but who are actually interested in physics or chemistry or molecular biology—who in high school hadn't advanced so far that they already differentiated into physicists or chemists or biologists.

What happens to students who come to college wanting to learn biochemistry? They find themselves first in a chemistry class with a hundred students with absolutely no interest in chemistry. All of those students drill a hole in the head of the instructors and each other to get the best possible grade because all they want is the grade. You teach these people later, and you realize that they are unteachable, to a first approximation. I have never failed as a teacher, except when trying to teach genetics to medical students.

I knew that reform was impossible. The word reform never passes my lips.

Instead, what I proposed was that we use this building, which could house as many as 15 or 16 new faculty, and use resources in the various fields to mount an alternative introductory curriculum in the first two years—at a very high level for students who are willing to do the math, students who want to learn computation—and offer incoming freshmen a choice. Students could learn in the usual way, with the standard, well-worked out curriculum. Math—you can go back 200 years, and you can't find a new thought. Chemistry is taught in exactly the same way that Linus Pauling prescribed in his book published, I think, in 1926.

Or the students could participate in this new experiment in which we'll teach computer science, physics, chemistry, and molecular biology corresponding to the introductory level in six semesters of work—four in the first year, two in the second year—and then major in any of those areas.

What we did was we had eight senior faculty, a sort of Noah's ark of science, sit every Monday at lunch for two to three hours and simply ask the following question for all of these fields at the most introductory level: Is this problem or this idea or this concept fundamental, or merely traditional? We collected all the fundamentals and did the best we could to make them into a coherent sequence.

We tell the students, “This is not the low-energy path to medical school.” When they come here, they have fear and trepidation, but I tell them, privately anyway, “Look, if you don't get too many Cs and you work in somebody's lab and do a good job, you'll get into any graduate school in the country. So stop worrying about the grades. If you get a B in everything that we do, you'll be a heroine or a hero.”

The adventurous students, the risk takers, come to us. They know how deadening it is to be part of premedical or preprofessional education. It is a mistake to underestimate the perceptiveness of children, as you know!

The breadth of the program has really been its great strength. Morale is very high. The other day, one of our students came to me and said she was thinking of majoring in computer science. She said, “I knew nothing, and I was terrified of it, and I had no idea. If I hadn't taken this thing, I would never have learned to program, but I love it!” Well, that's what we're here for!

The students had a final exam last spring, worth half their entire grade for the year, and all of them showed up in identical T-shirts they had designed. These students are really terrific. I haven't seen students like this since 20, 25 years ago at MIT. They are really turned on.

We have an endowment for five Lewis-Sigler Fellows. We search the country for the best experimentalists we can find, right out of graduate school, no post-doc. And we say, “Instead of being a post-doc, we'll give four benches, $250K a year for research, and you can stay for five years—but you have to teach the lab part of the undergraduate curriculum.” And they are fantastic.

**JG:** Sounds like you've recreated your old job at MIT! What about the graduate program?

**DB:** The graduate school is a more difficult problem, and it's a problem we haven't solved—yet—but we're working on it. We started this Quantitative and Computational Biology Program. This is being led by Leonid Kruglyak, who graduated from Princeton *summa cum laude* in physics. So the idea is that we are going to do exactly the same thing that we did with the undergraduates, in a way. All the participating departments, of which there are many, are going to admit a bunch of students who might be interested in the other field, and we'll try to teach them a few things together.

We have one such course called Method and Logic in Quantitative Biology, and we teach the classic papers, many of which are forgotten because they can't be taught now because people don't understand the math. You may remember reading Luria and Delbruck, and you may remember that nobody understood Luria and Delbruck because they didn't have the math—and these were MIT graduate students! Poisson distribution—don't bother me.

**JG:** The teaching enterprise is the big glue that is holding this institute together.

**DB:** And that's part of why they hired me, because they understood that eating lunch together was probably not going to be good enough to actually get people to talk to each other seriously. People need to have some common task that is orthogonal to their own research, and eventually, before you can turn around, they are doing stuff together.

This endeavor is not a solo endeavor of mine. Bill Bialek in physics and in the Lewis-Sigler Institute is the architect of the syllabus itself, and the main lecturer in the first semester. The laboratory parts of the freshman course were designed and run by two fellows—Maitreya Dunham, a geneticist, and Will Ryu, a physicist. These fellows are terrific. I'm organizing everything so that by the time I retire in seven years, everything will run on younger people, and they won't actually need me. I'm no spring chicken!

**JG:** Let's talk a bit about your roots. Your younger brother Leon is a well-known conductor, and I read somewhere that you also considered a career in music.

**DB:** Briefly. When I was in college, I did take harmony and counterpoint and was in the music scene. I was doing that and physics. But physics was taking most of my time.

What I was interested in was choral conducting. I was a singer. I sang in the Harvard glee club, and I was the rehearsal conductor sometimes. I still remember trying to get people to get the rhythm right in Stravinsky's *Oedipus Rex*. It's not that hard, but it's amateur singers. I don't have such a great voice, but I knew about music.

**JG:** Are you a baritone?

**DB:** Yes. In those days I could do bass; I had an acceptable F and an audible E, so it was enough. I was pretty good in the midrange. The high notes were not too challenging for me. I sang for many, many years. I sang, memorably, when Kennedy was killed. A huge pick-up group sang the Mozart *Requiem* in Symphony Hall. That was really an occasion.

**JG:** Did your parents sing?

**DB:** My mother's family was heavily into music in the old country in Poland. My grandfather was a great patron of the arts; he was a very rich man. There were many famous musicians who he sponsored to go to conservatory from that area of Poland. He survived the Holocaust, and I knew him.

My mother was a pediatrician. She was [Guido] Fanconi's assistant. She was in fact the first to show that cystic fibrosis is inherited.

**JG:** How did she show that?

**DB:** A family study in Switzerland during the war. Dorothy Anderson did the same thing at Columbia Babies Hospital, and our literature was all Anderson [because Fanconi published only in German]. It was Fanconi, of course, who discovered cystic fibrosis.

**JG:** I didn't know that.

**DB:** Failure to thrive, just like Fanconi's anemia.

Both my parents were physicians. My father was what you would call today a radiation oncologist. He was the first in this country to use betatrons.

**JG:** Was your father Swiss?

**DB:** Neither was Swiss. They were there in medical school [in Zurich], and they stayed there as residents and then the war broke out. He was from Poland also. Actually, the family comes from Odessa. The other side comes from Vilnius.

My mother was Fanconi's *oberartz*—the chief resident. Ania Wyszewianska, later Anne Botstein. When she came to this country, she was offered positions in various places, but she started to lose her hearing—she had Meniere's disease. She went into private practice. She worked in one of the first HMO's in New York, a place called the HIP at Montefiore hospital where she was Chief of Pediatrics for 25 years, so she was very successful. My father went academic and was a professor at Einstein. And he is very well known. His residents are all over the country.

**JG:** So you lived where?

**DB:** In the Bronx, in Riverdale, on the corner of the Hudson River, and on the Yonkers border.

**JG:** You were born in Switzerland?

**DB:** Yes.

**JG:** Did you have Swiss citizenship?

**DB:** No, there is no such thing as Swiss citizenship for foreigner Jews. Swiss citizenship is hereditary.

We were stateless, and then my parents got a visa for the United States in 1949. They had applied for an American visa in 1935 when they were married, but the Polish quota was full. Fourteen years later, one of their friends was at the US consulate, and they had just posted a list on the door of who had a visa, and they noticed that my parents were on the list. My parents had two weeks' notice to make up their minds as to whether they would come. And they did.

I remember that trip very well. I was seven. My mother had a cousin in New York, and they had letters of recommendation from their bosses in Switzerland who were very well known. My mother tells the story of taking the boards in pediatrics, and it was all “anonymous.” She had Nelson, of the famous textbook in Pediatrics, as her examiner, and they talked about polio and this and that, and then he says, “Well, you really know these statistics very well. I'm not supposed to do this, but I know you are not Fanconi—you must be Botstein!” And then they gossiped the rest of the time. It's the same thing as if Sydney Brenner tried to interview Ira Herskowitz.

My mother is still alive, and she lives in an apartment right next to my brother, and I see them nearly every week now. My brother and I have always been very close. He's the famous one. And deservedly so. He's a real innovator in teaching, too!

**JG:** Since I'm a human geneticist, I can't help but ask you about the genesis of the 1980 mapping paper with Davis and colleagues [in *American Journal of Human Genetics*].

**DB:** That's famously been written up a number of times. The account in Bishop and Waldholtz's book *Genome* is actually very accurate, as I recall.

**JG:** I want to hear it in your words.

**DB:** I was teaching with [Ron] Davis and [John] Roth, at Cold Spring Harbor, the bacterial genetics course in 1978. Jim [Watson], typically, was too cheap to allow us to get together. Roth was in Utah, I was in Boston, and Davis was at Stanford. So Roth finagled that we, Davis and I, would be outside readers for the training grant that Utah had. The genetics training program had a retreat in Alta.

We had to listen to all the talks, and one of the talks was by one of [Mark] Skolnick's students, the guy who did hemochromatosis, what's his name? If you're gonna make history you gotta look all this up. [Meanwhile, David does a quick PubMed search.] This kid got up and talked about how there were two ways to think about hemochromatosis and one of them involved linkage to HLA [human leukocyte antigen]. And the linkage to HLA would make hemochromatosis recessive. And you could build a reasonable model, and they had a likelihood score for that possibility.

Kerry Kravitz! 1978!

There were, at Utah, a lot of hard heads, mainly immunologists, and these immunologists said, “This is bull; it's got to be physiological because HLA affects all this other stuff, and you guys are talking through your hats.” They didn't like Skolnick's approach. They didn't like the whole idea.

Now, of course, it's 1978, nobody knows what a LOD [logarithm of the odds] score is, and the only person in the room who knows, besides Skolnick and Kravitz, is Botstein. So I find myself in the middle of this argument. This argument erupts, and I am defending this student because the student and I understand what he did, and nobody else does, and he has not managed to explain it. Skolnick isn't explaining it either. None of these guys are great explainers. It's a medical school, okay!

So finally I say something like, “Look there is nothing special about HLA. What's good about HLA is that it has many alleles, and because it has many alleles, you can tell if you have linkage, and if you have many multiallelic markers all over the genome, you can map anything!” And as soon as the words were out of my mouth, I look at Davis, Davis looks at me, and we both understand that of course there are such markers, and we could make a map of the human genome tomorrow.

**JG:** What kind of markers were you thinking of?

**DB:** We were thinking of two things, one of which would now be called SNPs [single nucleotide polymorphisms] and the other, which we expected would be more common, was insertion sequences, movements of transposons—because in yeast, that was what was going on. You see, Davis's part of this was TY1. Remember what my history is. In 1978, what was I known for?

**JG:** Uh, I give up.

**DB:** Tn10! We were one of the groups that discovered transposons in bacteria. Russell Chan, Nancy Kleckner—making mutations, mobilizing genes. It was a really big deal. So the first thing we thought of was, “Oh, there are going to be transposons all over the place!”

We were surprised to discover that the really good markers were all these CA repeats. We did not anticipate that. But I understood pretty much the whole thing, and then the rest of that meeting was—we sat in the bar, we drank, and we figured out how we could make a map of the human genome: how it would work and how many markers we would need. The whole 1980 paper. Everything in there was there in outline by the end of the day.

I went home and I explained it all to Maury Fox, and he understood why this was interesting, and he understood the math. And I persuaded Ray White to look for polymorphisms because he had been doing this transposon jumping around business in introns and ribosomal DNA in *Drosophila*. And it wasn't going anywhere. He had the right technology. And then he and Arlene Wyman found what is now called D14S1, and we were off.

What was really noticeable at the time was that the human geneticists didn't get it. At all. At all, at all, at all. It took a really long time. Skolnick was beating the drum. In 1983, I went to the ASHG [American Society of Human Genetics] meeting, and I gave this long discussion of how it all would work, and I had to explain Southern blot and this and that. I went to NIH [National Institutes of Health] and tried to get money, and Ruth Kirschstein looked at me and said, “We don't do things like that.” That was the origin of her opposition to the genome project, I'm sure.

Then [Jim] Gusella did his number [mapped Huntington disease]. On the one hand, it gave the idea some credibility; on the other, it did everybody a huge disservice, because people started to look with random markers. And the statistics!

**JG:** They were so lucky; it was the twelfth marker!

**DB:** The eighth! The eighth! The prior probability of finding it in eight markers was zero! I knew that!

**JG:** Well, for once, ignoring the math paid off!

**Figure:**
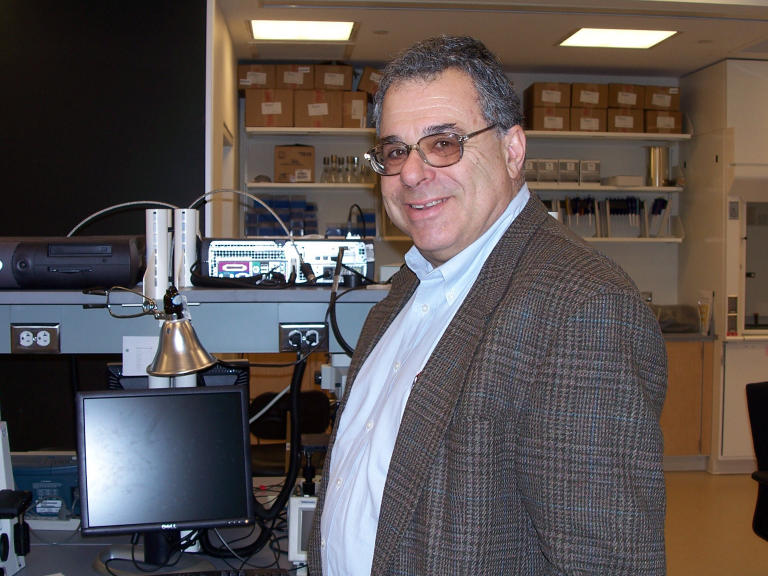
David Botstein

